# Effect of ethanol extract of nigella sativa L seeds and propofol on BDNF protein level as neuroplasticity and neuroprotection of traumatic brain injury in rats

**DOI:** 10.12688/f1000research.142054.1

**Published:** 2024-04-15

**Authors:** Kulsum Kulsum, Syahrul Syahrul, Kartini Hasbalah, Ummu Balqis

**Affiliations:** 1 Doctoral Program, Faculty of Medicine, Universitas Syiah Kuala, Banda Aceh, Aceh, 24415, Indonesia; 2 Anesthesiology and Intensive Therapy, Rumah Sakit Umum Daerah Dr Zainoel Abidin, Banda Aceh, Aceh, 24415, Indonesia; 3 Anesthesiology and Intensive Therapy, Faculty of Medicine, Universitas Syiah Kuala, Banda Aceh, Aceh, 23111, Indonesia; 4 Neurology, Rumah Sakit Umum Daerah Dr Zainoel Abidin, Banda Aceh, Aceh, 24415, Indonesia; 5 Neurology, Faculty of Medicine, Universitas Syiah Kuala, Banda Aceh, Aceh, 23111, Indonesia; 6 Pharmacology, Faculty of Medicine, Universitas Syiah Kuala, Banda Aceh, Aceh, 23111, Indonesia; 7 Pathology, Faculty of Veterinary, Universitas Syiah Kuala, Banda Aceh, Aceh, 23111, Indonesia

**Keywords:** BDNF, Nigella Sativa L, Propofol, Traumatic brain injury

## Abstract

**Background:**

Traumatic brain injury (TBI) is a change in brain function or evidence of brain pathology caused by external mechanical forces. Brain Derived Neurotrophic Factor (BDNF) is a neurotropin that functions as a neuron protective. Nigella sativa L is reported to have an antioxidant effect, administration of Nigella Sativa L to rats treated with ischemia-reperfusion brain injury. Propofol is an anesthetic agent frequently used intravenously in the management of TBI. The effect of propofol on brain tissue after TBI may be neuroprotective. We aimed to compare the potential of Nigella sativa L and propofol as neuroplasticity and neuroprotection in rats with TBI.

**Methods:**

This was a laboratory experimental animal model with the post-test only control group design, namely measuring the effect of treatment by comparing the five groups of rats consisting of 30 rats. BDNF levels in rat brain tissue were collected at day 7 of treatment and measured by ELISA.

**Results:**

The average BDNF protein levels per group, namely G1 (221,243 pg/mL), G2 (172,139 pg/mL), G3 (255,483 pg/mL), G4 (227,089 pg/mL), and G5 (272,603 pg/mL) respectively. Based on the ANOVA statistic, p-value = 0.032 (there was a significant difference between groups), with the Levene Test (0.077) or having variance between the same groups, sequentially the difference in average BDNF protein levels of the five groups is G5>G3>G4>G1>G2, meaning that the combination of Nigella sativa and propofol has more potential to increase BDNF protein levels than Nigella sativa, and Nigella sativa has more potential than propofol.

**Conclusion:**

We concluded that both nigella sativa and propofol have the potential to increase BDNF protein levels. Nigella Sativa L had a better effect than propofol in repairing damaged neuron cells (neuroplasticity) and increasing BDNF protein levels (neuroprotection) for 7 days of administration in rat traumatic brain injury.

## Introduction

In the community, there are health problems such as traumatic brain injury (TBI) causing neurocognitive and psychological disorders such as impaired attention, inability to form visuospatial associations, poor executive function, and depression ^1^^,^^2^ A total of sixty-nine million (95% Confidence Interval (CI) 64-74 million) people are estimated to experience TBI every year worldwide. The highest proportion of TBI due to traffic accidents occurred in Southeast Asia and Africa (both around 56%) and the lowest in North America (25%). The incidence of TBI is also highest in Southeast Asia (1.5% of the population per year) and Europe (1.2%). The overall incidence of TBI per 100,000 people is highest in North America and Europe with 1299 cases in America and 1012 cases in Europe and the least in the Eastern Mediterranean (897 cases) and Africa (801 cases). ^3^

Brain Derived Neurotrophic Factor (BDNF) is a neurotropin that is useful as a neuroprotective. BDNF is an important molecule in brain plasticity. The increase in BDNF is related to the progress of improvement in nerve function. However, after ischemia of the brain injury occurs, BDNF will decrease either spontaneously or by induction of rehabilitation so that neuroplasticity or repair of nerve cell function will change. ^4^^,^^5^ The central and peripheral nervous systems are the working areas of BDNF. BDNF also stimulates the growth of new neurons and their ability to survive. The hippocampus, cortex, and frontal lobe in the base of the brain are where BDNF works actively. ^4^^–^^7^

Nigella sativa oil is reported to have antioxidant, antihistamine, anti-inflammatory, immunomodulatory, antitussive, antidiabetic, antihypertensive, antipyretic, analgesic, anticancer, and antimicrobial effects. ^8^^–^^14^ Protecting nerve cells from damage due to disease processes or chemicals has also been widely reported. ^15^^–^^18^ In a study conducted by Zadeh *et al.*, Nigella sativa significantly increased BDNF levels in depressed patients. This study indicates the importance of administering Nigella sativa to cure depression. This study was statistically significant comparing Nigella sativa with placebo. In this study, decreased BDNF increased the risk of Alzheimer’s disease, stress, depression, and anxiety. There have been many reports that state BDNF levels can be a predictor of a neuropsychiatric disease. ^7^ BDNF functions as a growth and development protein in the central nervous system and peripheral nervous system and is very active in the hippocampus and parts of the brain cortex. ^7^^–^^9^

The results of a study by Hosseinzadeh *et al.*, stated that administration of Nigella sativa L oil to rats treated with ischemia-reperfusion brain injury in the hippocampus. The active ingredient of Nigella sativa L oil is thymoquinone. Timoquinone, the main constituent of essential oil from Nigella sativa seeds, is reported to have antioxidant properties that can increase protection and resistance to oxidative stress. ^18^ Another study from Abbasnezhad *et al.* stated that thymoquinone as an antioxidant can significantly reduce oxidative stress which damages hippocampal neurogenesis. A previous study has been conducted on Nigella Sativa L as a potential antioxidant because its chemical compounds have the potential to be effective in capturing OH radicals during non-enzymatic lipid peroxidation and deoxyribose degradation. ^12^

Propofol is a fast-acting intravenous induction anesthetic agent and is widely used in the treatment of TBI because of its good induction ability. ^19^ BDNF is useful in reducing the density of propofol synapses followed by activation of growth factor receptors p75NTR and RhoA, namely actin depolymerization, loss of microtubules, and disruption of axon transport resulting in inhibition of BDNF traffic. ^20^^,^^21^ Propofol is an intravenous anesthetic agent used as an anesthetic induction agent and is also widely used to sedate patients in intensive care units. Many preliminary studies suggest that propofol has a neuroprotective effect against brain ischemia. ^22^ However, in the study of Kotani *et al.*, the evidence that propofol can reduce ischemic brain damage is inconclusive, and further research is needed. ^23^

Yusriyani’s research found that there was an effect of propofol on the percentage of caspase 3 expression in the hippocampus in mice that had experienced traumatic brain injury. This study used different doses of propofol starting from doses of 10 mg, 25 mg, and 50 mg/kg body weight intravenously. The results of the study found significant differences between treatment groups. The most effective dose to reduce the percentage of caspase 3 expression in the hippocampus in mice is a dose of 50 mg/kg body weight intravenously. Of the statement above, the author’s interest arose to research the effect of Nigella sativa seed extract and propofol on BDNF as neuroplasticity and neuroprotection of traumatic brain injury in rats (Rattus novergicus). This study will later assess the amount of BDNF levels in the cerebrospinal fluid using the Enzyme-linked immunosorbent assay (ELISA) method in the treatment group compared to the control group. ^24^ The results of research on propofol in vivo and in vitro have not been able to explain with certainty the mechanism of propofol as neuroplasticity and neuroprotection of the brain. Therefore researchers want to analyze the effect of giving propofol to BDNF on traumatic brain injury so that it can be used as the basis for an effective treatment.

## Methods

### Study design and subjects

This was a laboratory experiments animal model with the post-test only control group design, namely measuring the effect of treatment by comparing the five groups. G1 = Negative control (without TBI treatment), G2 = Positive control (TBI without drug administration), G3 = TBI with Nigella sativa 500 mg/kgBW P. O. administration, G4 = TBI with Propofol 10 mg/kgBW I. M. administration, G5 = TBI by administering a combination of Nigella sativa 500 mg/kgBW P. O. and Propofol 10 mg/kgBW I. M. The data was collected from April 2023 to May 2023.

The subjects of this study were Rattus novergicus rats, adult males, aged 4-8 weeks, weighing 150-200 grams, obtained from the Faculty of Veterinary Medicine, University of Syiah Kuala. The sample selection technique used in this study is the Federer formula.

Laboratory data including BDNF protein level were collected on day 8 of the TBI treatment. In total, 30 rats were selected during the study period.

### Data collection

**Preparation of animal model**

Prior to the treatment, the fur was assigned a specific number for use in the straightforward randomization procedure. This hair shaving can cause wounds but this has to be anticipated by shaving carefully and using a very sharp razor to avoid damage to the animal’s skin. Following this, 10 days period of adaptation took place, homogenization was conducted in the cage. The temperature in the cage was adjusted to room temperature, and the rats were fed with BR I on a daily basis.

**Administration of TBI**

The Feeney weight drop mode was carried out using male Rattus norvegicus rats. These rats were shaved and had a 4 mm incision made on their right frontoparietal area to expose the bone. The skin opening’s center was positioned 1.5 mm posterior to the bregma and 2.5 mm lateral from the midline. Subsequently, the rats were dropped from a 1 meter height, with a weight diameter of 2.5 mm. Earlier research had previously conducted initial trials to create experimental animal models. The results from these earlier studies with the Feeney TBI model demonstrated an increase in inflammatory levels of TF Alpha and elevated MDA ischemic levels.

**Surgical procedure**

On the 7th day following the rats exposure to TBI, the rats were euthanized using the cervical dislocation technique. Subsequent to euthanasia, craniotomy was performed on the rats, allowing for the extraction of brain tissue. The collected brains were weighed and their weights were documented. The entirety of the process was executed by the same operator utilizing microsurgical tools.

**BDNF protein level with ELISA method**

Brain Tissue Protein Isolation. Fresh rat brain tissue was cut into small pieces and then rinsed with cold PBS (0.01 M, pH 7.4) to remove blood that was carried away during the collection process. The tissue was weighed ± 0.1 gram and added assay buffer with a ratio of 1:9 (w/v) then homogenized using a paste followed by a freeze-thaw cycle to optimize the destruction of brain tissue cells. The rat brain tissue homogenate was then centrifuged at 5,000×g at 40C for 10 minutes.

BDNF levels in rat brain tissue were measured using a commercial kit (Elabscience Rat BDNF Catalogue number E-EL-R1235) using standard concentrations of 0, 31.25, 62.5, 125, 250, 500, 1000, and 2000 pg/ml. The initial stage of the ELISA examination begins with adding 100 ul of sample and standard into each well plate that has been coated with a specific antibody. The Elisa plate was then incubated at 37 ^o^C for 90 minutes. The sample/standard solution on the Elisa plate was then removed and then 100 ul of Biotinylated detection AB solution was added and incubated at 37°C for 60 minutes. Next, the Elisa plate was washed 3 times using an Elisa washer followed by the addition of 100 ul HRP conjugate working solution and incubated at 37°C for 30 minutes. The ELISA well plates were then washed again 5 times and then 90 ul of reagent substrate was added to each Elisa well plate. At this stage, a color change reaction occurs after the Elisa plate is incubated for 15 minutes. To stop the color change reaction, 50 ul stop solution was added to each Elisa well plate, the addition of the solution would change the color of the reaction from blue to yellow. The color was then measured at a wavelength of 450 nm using a microplate spectrophotometer. Step by step description of BDNF procedures can be found on Protocols.io, DOI: dx.doi.org/10.17504/protocols.io.ewov1q3w2gr2/v1.

### Statistical analysis

Data analysis in this study used one-way ANOVA (Analysis of Variance) to test the hypothesis of the study with a significance level of 0.05 (α = 5%). Before data analysis, the ANOVA prerequisite test was carried out, namely the normality test and homogeneity test which were calculated with the help of a software application by using SPSS (IBM, New York, US). The normality test was carried out on the residual results of the ANOVA model using the Shapiro-Wilk test because the number of samples was < 50.

### Ethics approval

This study was approved by the Veterinary Research Ethics Committee of the Faculty of Veterinary, Universitas Syiah Kuala, Banda Aceh, Indonesia, No. 208/KEPH/III/2023.

## Result

The results of the ELISA to determine BDNF protein levels in rat brain tissue can be seen in Table 1.

**Table 1.** BDNF protein levels in brain tissue using the ELISA method.

Groups	Mean	Std. Deviation	Median	Minimum	Maximum	p *	p **
G1	221.24	52.31	197.47	181.90	316.77	0.054	0.032
G2	172.14	20.49	172.76	141.17	198.56	0.989	
G3	255.48	72.50	277.08	156.26	333.28	0.371	
G4	227.09	38.09	222.94	188.89	270.05	0.121	
G5	272.60	64.98	258.54	198.30	377.30	0.764	

* Shapiro-Wilk test.

** ANOVA test.

The data shown in Table 1. shows data on BDNF protein levels in rat brain tissue using the ELISA method in all groups that appear to be normally distributed. G2 rats had the lowest level of BDNF protein in brain tissue compared to the other groups, while G5 had the highest score. The results of analysis of BDNF protein levels in rat brain tissue using the ELISA method between groups showed that there were significant differences. Post hoc analysis for analysis between groups of rats is presented in Table 2 as follows:

**Table 2.** Results of Post hoc analysis of BDNF protein levels in rat brain tissue using the ELISA method.

Groups	G1	G2	G3	G4	G5
G1		0.122	0.274	0.850	0.106
G2	0.122		0.012	0.085	0.003
G3	0.274	0.012		0.363	0.581
G4	0.850	0.085	0.363		0.150
G5	0.106	0.003	0.581	0.150	

**Figure 1.**
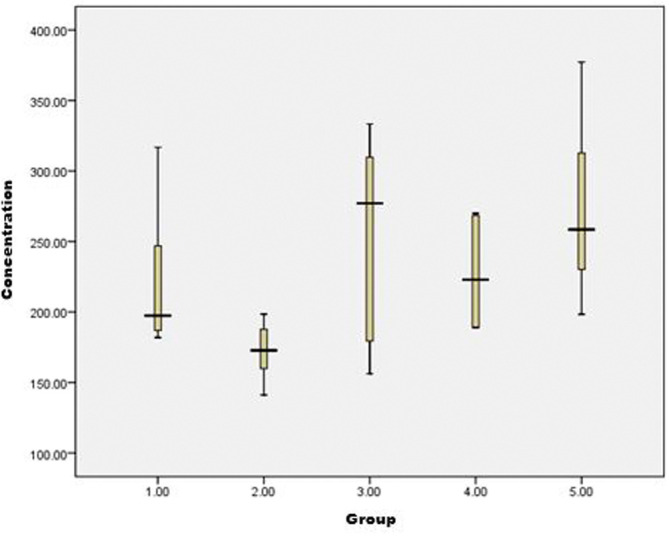
Boxplot data of BDNF protein levels in rat brain tissue using the ELISA method.

Based on the results of the analysis between groups, the levels of BDNF protein in brain tissue that showed a significant difference (P < 0.05) were between G2 compared to G3 and G5, while the levels of the other groups did not differ significantly. It can be concluded that the BDNF protein levels in the brain tissue of rats using the ELISA method in positive control rats were significantly lower when compared to the group of rats given Nigella sativa 500 mg/kgBW P. O. and the group of rats given a combination of Nigella sativa 500 mg/kgBW P. O. and Propofol 10 mg/kgBW I.M. Based on Figure 1, the longest interval of BDNF protein levels is obtained at G3 and the shortest at G2. The highest BDNF protein level was found in G5 with a level of 377,300, followed in G3 with a total level of 333,279 and the lowest was in G2 with a BDNF protein level of 141,146. However, qualitatively based on Figure 1 it can be seen that the highest peak of the median is G3, followed by G5, G4, G1, and G2 sequentially.

The results obtained based on this test were at a significance level of 1% and based on the output of the normality test, the Shapiro-Wilk values were all greater than 0.01 ( Table 3), so it can be concluded that the residual values are normally distributed. The homogeneity test was carried out using the Levene test ( Table 4). The results obtained are as follows: with a significance level of 1% and based on Levene’s test output, a p-value of 0.077 is obtained. This value is greater than 0.01 so it can be concluded that the variance of the variables is the same or homogeneous. Because the normality and homogeneity assumptions have been fulfilled, the one-way ANOVA test can be carried out. The results of one-way ANOVA calculations in this study can be seen in Table 5.

**Table 3.** Shapiro-Wilk test of normality.

Tests of normality							
	Group	Kolmogorov-Smirnov ^a^			Shapiro-Wilk		
		Statistic	Df	Sig.	Statistic	df	Sig.
Concentration	1	.309	6	.075	.796	6	.054
	2	.110	6	.200 ^*^	.990	6	.989
	3	.212	6	.200 ^*^	.900	6	.371
	4	.235	6	.200 ^*^	.836	6	.121
	5	.202	6	.200 ^*^	.953	6	.764

^a^ Lilliefors significance correction.

* This is a lower bound of the true significance.

**Table 4.** Lavene test of homogeneity of variances.

Concentration			
Levene statistic	df1	df2	Sig.
2.403	4	25	.077

**Table 5.** One way ANOVA.

ANOVA					
Concentration					
	Sum of squares	df	Mean square	F	Sig.
Between Groups	35382.109	4	8845.527	3.140	.032
Within Groups	70426.708	25	2817.068		
Total	105808.816	29			

Based on Table 5 p-value <0.05 (p-value = 0.000) and F count (3.140) > F table (2.98) then H0 is rejected and H1 is accepted. This means that there is a difference in the average results of the ELISA examination in each group. Based on the ANOVA statistic, the test results obtained were p = 0.032 (there was a significant difference between groups), with the Levene Test (0.077) or having variance between the same groups, Post hoc test with LSD test showed G1 did not have a significant difference with other groups, G2 was significantly different G3 and G5 were significantly different, G3 was significantly different from G2, G4 was not significantly different from all groups, and G5 was significantly different from G2. Then a Post hoc test was carried out as a follow-up test from the ANOVA test to find out which variable had the average difference. significant. Further test results with the Post hoc test method can be seen in Table 6.

**Table 6.** Post hoc tests.

Multiple comparisons							
Dependent variable:							
	(I) Group	(J) Group	Mean difference (I-J)	Std. Error	Sig.	95% Confidence Interval	
						Lower bound	Upper bound
LSD	1	2	49.10350	30.64348	.122	-14.0079	112.2149
		3	-34.24017	30.64348	.274	-97.3516	28.8713
		4	-5.84567	30.64348	.850	-68.9571	57.2658
		5	-51.36033	30.64348	.106	-114.4718	11.7511
	2	1	-49.10350	30.64348	.122	-112.2149	14.0079
		3	-83.34367 ^*^	30.64348	.012	-146.4551	-20.2322
		4	-54.94917	30.64348	.085	-118.0606	8.1623
		5	-100.46383 ^*^	30.64348	.003	-163.5753	-37.3524
	3	1	34.24017	30.64348	.274	-28.8713	97.3516
		2	83.34367 ^*^	30.64348	.012	20.2322	146.4551
		4	28.39450	30.64348	.363	-34.7169	91.5059
		5	-17.12017	30.64348	.581	-80.2316	45.9913
	4	1	5.84567	30.64348	.850	-57.2658	68.9571
		2	54.94917	30.64348	.085	-8.1623	118.0606
		3	-28.39450	30.64348	.363	-91.5059	34.7169
		5	-45.51467	30.64348	.150	-108.6261	17.5968
	5	1	51.36033	30.64348	.106	-11.7511	114.4718
		2	100.46383 ^*^	30.64348	.003	37.3524	163.5753
		3	17.12017	30.64348	.581	-45.9913	80.2316
		4	45.51467	30.64348	.150	-17.5968	108.6261

* The mean difference is significant at the 0.05 level.

The results of the ANOVA test were p = 0.032 (there were significant differences between groups), with the Levene Test (0.077) having the same variance between groups. The post hoc test with the LSD test showed that Group 1 had no significant difference from the other groups, group 2 was significantly different from Groups 3 and 5; Group 3 was significantly different from Group 2; Group 4 was not significantly different from all groups; and Group 5 is significantly different from group 2.

Table 6 shows that with a significance level of 5% (α = 5%), the average G5 is significantly different from G2 (p = 0.003, p < 0.05). The mean of G3 was significantly different from that of G2 (p = 0.012, p < 0.05), and the mean of G3 was not significantly different from that of G4 (p = 0.363, p > 0.05). Based on these results it can also be seen that the average difference between G5 and G2 is 100.46383 (the BDNF protein content of G5 is 100 points greater than G2), the average difference between G5 and G4 is 45.51467 (G5 is 45 points greater than G4), followed by the average difference between G5 and G3 is 17.12017 (G5 is 17 points bigger than G3), while the average difference between G3 and G4 is 28.39450 (G3 is 28 points bigger than G4). Thus sequentially the difference in average BDNF protein levels from the five groups is G5 > G3 > G4 > G2, meaning that the combination of Nigella sativa and propofol has more potential to increase BDNF protein levels than Nigella sativa, and Nigella sativa has more potential than propofol.

## Discussion

### BDNF protein level

The present study was conducted on 30 Rattus novergicus rats treated with BDNF examination after 7 days of TBI. Within seven days after administration of Nigella sativa and propofol, changes in the levels of the BDNF gene were found. This present study has comparable results with the results of research conducted by Hutagalung, ^25^ this present study was carried out with Nigella sativa extract, in contrast to Hutagalung using curcumin. Hutagalung’s study proves that the administration of curcumin from turmeric extract can increase mBDNF expression in rats that have experienced traumatic brain injury. Rats model were divided into 3 groups and each group consisted of 11 rats. All rats were observed after 24 hours after being treated for traumatic brain injury. In the Hutagalung study, it was found that curcumin from turmeric extract orally can increase mBDNF expression in brain cells that have experienced traumatic head injury. ^25^

In contrast to a study conducted by Zhou *et al.*, using 120 Sprague-Dawley rats to look at the mechanism of the effect of propofol on cognitive function. ^26^ As a comparison, a BDNF study in humans was conducted by Dharmajaya. Based on the results of statistical analysis on 16 samples of patients with head injuries, significant values were obtained on examination of BDNF levels. There was an increase in BDNF in the first 24 hours after TBI, then it increased at 48 and 72 hours after TBI and decreased in levels 120 hours after injury. ^6^

BDNF is a neurotrophic factor that helps the maturation, differentiation, and survival of neurons in the nervous system. In addition, in bad conditions, BDNF can show neuroprotective effects in the form of glutamatergic stimulation, hypoglycemia, cerebral ischemia, and neurotoxicity. BDNF functions to stimulate and control neurogenesis. BDNF can be found in most areas of the brain such as the cortex, hippocampus, mesencephalon, hypothalamus, olfactory bulb, basal forebrain, brainstem, and spinal cord. BDNF levels in neurodegenerative diseases such as multiple sclerosis (MS), Parkinson’s, and Huntington’s disease. In addition, BDNF also plays a role in the process of homeostasis. ^5^^–^^7^^,^^27^ The rat’s BDNF gene consists of eight exons, each with its promoter, and one 3’ exon encoding the active BDNF protein. Rat BDNF is very similar in structure to human BDNF. Exons I-III are located mainly in the brain, whereas exons IV are identified in the lung and heart, resulting in the transcription of eight different mRNAs. BDNF mRNA is highly expressed in the brain, according to in situ hybridization investigations. BDNF expression levels are very low during fetal development, increase after birth, and decrease in adults. ^28^

This present study is in line with the results of research by Wu *et al.* Based on the research results of Wu A, *et al.*, there is an increase in BDNF levels in head injuries which serves as an indicator of healing and as a sign of an increase in the ability to form nerve cells and a reduction in the impact of cognitive decline. In this study, the researchers showed that the presence of endogenous BDNF and pro-BDNF were also replaced by additional nutrition. ^29^

The conclusion from the study of Elfving *et al.* shows that the determination of BDNF in plasma samples is affected by temperature, caution should be exercised when interpreting the results where BDNF levels have been determined in plasma samples. Comparing BDNF measurements in mouse brain and mouse serum led to the conclusion that the BDNF ELISA kit has better accuracy, yield, and inter- and intra-test variation in serum samples compared to brain samples. ^30^

Research by Hicks *et al.* states that BDNF and its receptor, trkB, can provide trauma-related neuroprotection in the central nervous system. However, several other studies suggest that BDNF is a contributing factor to neurodegenerative events in trauma. This study aims to determine the role of BDNF in neuroprotection. Research conducted by Hicks *et al.* Regarding neurotrophins, especially BDNF in head injuries, it was found that the time for an increase in BDNF was between the first 24−72 hours post-traumatic. ^31^

Juananda *et al’s* research states that BDNF is believed to have a neuroprotective effect involved in synaptic plasticity and cognitive function. This function is mediated by intracellular signaling cascades via the mitogen-activated protein kinase (MAPK) and PI3K pathways. In the cerebellum, BDNF is involved in granular cell neurogenesis. BDNF can also increase neuronal growth, maturation, and differentiation of nerve cells and can protect cerebellum granular cells from apoptosis due to loss of glucose or hypokalemia. ^28^

Based on the results of a study conducted by Bathina *et al.*, BDNF is a neurotrophin that has a neuroprotective effect against ischemic brain injury. BDNF increases in the area around the lesion which is related to the progress of nerve function improvement. However, after brain ischemia injury occurs it causes a decrease in the level of BDNF which results in changes in the ability of neuroplasticity or repair of nerve cell function, spontaneously or by inducing rehabilitation BDNF is an important molecule in brain plasticity. ^4^

### Effect of nigella sativa

In present study, from the results of statistical analysis on the ELISA in determining the BDNF protein level, it can be stated that the peak potential for increasing BDNF in all treatment groups is G3 > G5 > G4 > G1 > G2 respectively, meaning that administration of Nigella sativa L extract is better at increasing BDNF levels than giving the combination of Nigella sativa L + propofol, and propofol alone so that it can be stated that giving Nigella sativa L extract increases the potential for neuroplasticity.

The results of this study are in line with the research of Nazwar *et al.* A study conducted by Nazwar *et al.*, found that giving Nigella sativa (black cumin) extract to rats with head trauma increased BDNF levels. In experimental rats with head injury, injection of black cumin extract reduced apoptosis, although not significantly. In experimental rats with head injury, black cumin extract treatment led to an association between BDNF and apoptosis. Further research is needed to determine the mechanism or method used by black seed extract to increase BDNF expression. It is necessary to carry out pharmacological studies of various black cumin extraction methods to increase understanding of thymohydroquinone. To learn more about thymohydroquinone, it is necessary to do pharmacological research on black cumin extraction methods. This is because the administration of black cumin extract orally to humans requires clinical trials. ^32^

In a study conducted by Zadeh *et al.*, Nigella sativa significantly increased BDNF levels in depressed patients. This study indicates the importance of administering Nigella sativa to cure depression. This study was statistically significant comparing Nigella sativa with placebo. In this study, decreased BDNF increased the risk of Alzheimer’s disease, stress, depression, and anxiety. There have been many reports that state BDNF levels can be a predictor of a neuropsychiatric disease. BDNF functions as a growth and development protein in the central nervous system and peripheral nervous system and is very active in the hippocampus and parts of the brain cortex. ^7^

This present study was carried out with Nigella sativa extract, in contrast to Motaghinejad *et al’s* research using curcumin. Motaghinejad *et al’s* study also investigated the role of curcumin in vivo in protecting rat hippocampal cells against alcohol-induced neuroapoptosis, oxidative stress, neuroinflammation, and reduced cell density and aimed to study the potential involvement of phospho-CREB-BDNF signaling in curcumin-mediated neuroprotection. ^33^ It could be argued that Nigella sativa has the same neuroprotective potential as curcumin.

This present study is similar to that of Wu *et al.* because Nigella sativa contains the active substance Docosahexaenoic Acid (DHA). According to the research of Wu *et al.*, Docosahexaenoic Acid (DHA) is one of the main omega-3 polyunsaturated fatty acids in the brain which plays an important role in neurological development, maintenance of learning, and memory, and increases fluidity and signal transduction function and regulates gene expression. The DHA supplement diet is expected to protect against reduced plasticity and side effects that occur after injury. Wu *et al’s* research concludes that supplementation of omega-3 fatty acids can restore cognitive function and normalize the action of BDNF, synapsin I, and CREB as well as oxidative damage after traumatic brain injury. ^34^

Slightly different from this present study. If the intake of certain nutrients in the form of Nigella sativa was given in this study, then in the study by Oliveira *et al.* the subjects in this study were not given dietary rules with special replacements. for healing to continue. Several studies have shown that BDNF is a key molecule for neuroplasticity. Oliveira *et al’s* study showed that increased BDNF in the central nervous system is a particular response to inflammatory stimuli, namely as a special neuroprotector. ^35^

### Effect of propofol

In this present study, propofol (G4) was also able to increase BDNF protein levels in the brains of traumatic brain-injured rats compared to the positive control group (G2), although its potency was lower than that of Nigella sativa L (G3). TBI increases oxidative stress, and reduced levels of BDNF, synapsin I, and cAMP. Responsive Element-Binding Protein (CREB) in the brain. TBI causes a decrease in BDNF levels, but within 48 hours the BDNF levels will rise. BDNF stimulates the mitogen-activated protein kinase/extractcellular signal-regulated kinase (MAPK/ERK), phosphoinositide-3 kinase (PI3K), and phospholipase C (PLC)-gamma pathway through activation of the receptor tyrosine kinase B (TrKB), a high-affinity receptor for BDNF. ^26^

The present study has produced evidence that propofol may exert a neuroprotective effect. In line with the results of Kotani *et al’s* study, propofol has been reported to have a neuroprotective effect that can reduce bleeding and intracranial pressure. ^23^

In line with the present study, a study conducted by Jacques *et al.* stated that propofol is a highly hydrophobic intravenous hypnotic in the form of a lipid emulsion. Like many intravenous anesthetics, propofol can decrease brain oxygen consumption, decrease glutamate release, and modulate GABA-A receptor activity. In vitro, propofol reduces oxidative stress. ^36^

The study by Hausburg *et al.*, found that in a rabbit model of cerebral IRI, propofol increased plasma SOD activity, which was associated with increased neuronal survival. Furthermore, in the rat model of IRI, ischemic infarct size was reduced by 21% in animals anesthetized with propofol. In addition, the neurological outcome in the rat model of incomplete ischemia was significantly improved in the rats anesthetized with propofol. ^37^

White *et al.*’s study demonstrated that propofol exhibits a neuroprotective effect in improving neurological function in brain-traumatized rats, which is independent of intralipid. In addition, propofol treatment can effectively reduce the expression of pro-inflammatory cytokines in the injured cortex of late-phase brain trauma. ^38^ The neuroprotective effects of propofol have long been recognized in animal studies such as ischemia/reperfusion models and brain trauma models, which have largely focused on the mechanism complex of propofol. to relieve brain edema, neuronal apoptosis, inflammatory response, and so on. ^39^ Through a rat model of ischemia, Shi’s research showed that propofol reduces inflammatory reactions and brain damage in focal cerebral ischemia in rats. ^40^

The study by Zhou *et al.* showed that the neuroprotective effect of propofol on brain trauma-induced brain damage was attributed to its anti-inflammatory properties. However, this study did not demonstrate the effect of propofol on cytokine expression in late-phase brain trauma. This study, to our knowledge, is the first time to show that the increased expression of IL-1β, IL-6, and TNF-ɑ genes on days 3 and 7 after brain trauma can be inhibited remarkably by propofol administration. Changes in IL-1β expression were most prominent among the three cytokines after propofol treatment on days 1 and 3. These results suggest that IL-1β may play a more important role than IL-6 and TNF-α under propofol administration after brain trauma. The results of this study provide new evidence for overcoming cytokine changes in brain-traumatized rats by time-delayed administration of propofol after brain trauma, Regarding the effect of propofol on neurologic function, in a model of cerebral ischemia, propofol was found to improve neurologic function. ^41^

## Conclusions

We concluded that both nigella sativa and propofol have the potential to increase BDNF protein levels. Nigella Sativa L had a better effect than propofol in repairing damaged neuron cells (neuroplasticity) and increasing BDNF protein levels (neuroprotection) for 7 days of administration in rat traumatic brain injury.

## Data Availability

Zenodo: BDNF Dataset, https://doi.org/10.5281/zenodo.10276418. ^42^ Zenodo: ARRIVE checklist for “Effect of ethanol extract of nigella sativa L seeds and propofol on BDNF protein level as neuroplasticity and neuroprotection of traumatic brain injury in rats, https://doi.org/10.5281/zenodo.10276505. ^43^ Data are available under the terms of the Creative Commons Attribution 4.0 International license (CC-BY 4.0).

## References

[1] Sastrodiningrat AG : Traumatic brain injury: primary brain damage, secondary brain damage, management, and neuro critical care. *Book.* Medan: Sumatera Utara University Press;2012; pp.125–182.

[2] Das M Mohapatra S Mohapatra SS : New perspectives on central and peripheral immune responses to acute traumatic brain injury. *J.Neuroinflammation.* 2012;9(236):1–12. 23061919 10.1186/1742-2094-9-236 PMC3526406

[3] Dewan MC Rattani A Gupta S : Estimating the global incidence of traumatic brain injury. *J.Neurosurg.* 2018;1(1):1–18. 10.3171/2017.10.JNS17352 29701556

[4] Bathina S Das UN : Brain-derived neurotrophic factor and its clinical implications. *Arch. Med. Sci.* 2015;11(6):1164–1178. 10.5114/aoms.2015.56342 26788077 PMC4697050

[5] Salehi Z Mashayekhi F : Brain-derived neurotrophic factor concentrations in the cerebrospinal fluid of patients with Parkinson’s disease. *J. Clin. Neurosci.* 2009;16:90–93. 10.1016/j.jocn.2008.03.010 19017558

[6] Dharmajaya R : Brain Derived Neurotrophic Factor (BDNF) post-severe head injury as a predictor of disease course. *Indones. J. Clin. Pathol. Med. Lab.* 2014;21:61–66. 10.24293/ijcpml.v21i1.1261

[7] Zadeh AR Eghbal AF Mirghazanfari SM : Nigella sativa extract in the treatment of depression and serum Brain-Derived Neurotrophic Factor (BDNF) level. *J. Med. Res. Sci.* 2022;27(28):26–28. 10.4103/jrms.jrms_823_21 PMC9081508 35548175

[8] Azzubaidi MS Al-Ani IM Saxena AK : Prevention of brain hypoperfusion-induced neurodegeneration in rat’s hippocampus by black cumin fixed oil treatment. *IIUM Med. J. Malaysia.* 2018;17(1):1–4. 10.31436/imjm.v17i1.309

[9] Tavakkoli A Hosseinzadeh H : Nigella sativa L., and thymoquinone as neuroprotective antioxidants. *Chapter 21. Oxidative stress and dietary antioxidants in neurological disease.* 2020; pp.1–17.

[10] Landucci E Mazzantini C Buovicino D : Neuroprotective effects of thymoquinone by the modulation of ER stress and apoptotic pathway in in vitro model of excitotoxicity. *Molecules.* 2021;26:1–11. 10.3390/molecules26061592 PMC7998420 33805696

[11] Islam MH Ahmad IZ Salman MT : Neuroprotective effects of *Nigella sativa* extract during germination on central nervous system. *Pharmacogn. Mag.* 2015;11(42):182–190. 10.4103/0973-1296.157729 PMC4461959 26109765

[12] Abbasnezhad A Hayatdavoudi P Niazmand S : The effects of hydroalcoholic extract of *Nigella sativa* seed on oxidative stress in hippocampus of STZ-induced diabetic rats. *Avicenna J. Phytomedicine.* 2015;5(4):333. PMC4587602 26445713

[13] Marwan K Boom CE Ruslami R : Statins as brain protectors in traumatic brain injury. *J. Neuroanestesi Indones.* 2017;6(1):48–57. 10.24244/jni.vol6i1.39

[14] Istikomah I Purwanto B Ardiyanto TD : The effect of date palm juice and green tea supplementation on reducing plasma malondialdehyde (MDA) levels in children with thalassemia. *J. Kesehat Kusuma Husada.* 2018;162–167. 10.34035/jk.v9i2.275

[15] Isaev NK Chetverikov NS Stelmassok EV : Thymoquinone as a potential neuroprotector in acute and chronic forms of cerebral pathology. *Biokhimiya.* 2020;85(2):167–176. 10.1134/S0006297920020042 32093593

[16] Babar ZU Azad AK Sulaiman WMAW : Neuroprotective properties of *Nigella sativa (L.)* seeds extract in Sprague dawley rats model. Dhaka Univ. *J. Pharm. Sci.* 2018;17(1):113–121. 10.3329/dujps.v17i1.37127

[17] Asiei F Fazel A Rajabzadeh AA : Neuroprotective effect of *Nigella sativa* extract upon the hippocampus in PTU-induced hypothyroidism juvenile rats: a stereological study. *Metab. Brain Dis.* 2017;32:1755–1765. 10.1007/s11011-017-0025-1 28497360

[18] Hosseinzadeh L Monaghash H Ahmadi F : Bioassay guided isolation of neuroprotective fatty acid from *Nigella sativa L.* against 1-methyl -4-phenylpyridium-induced neurotoxicity. *Pharmacogn.Mag.* 2017;13:627–633. 10.4103/pm.pm_470_16 29200724 PMC5701402

[19] Woldegerima N Rosenblatt K Mintz CD : Neurotoxic propoerties of propofol sedasion following traumatic brain injury. *Crit. Care Med.* 2016;44(2):455–456. 10.1097/CCM.0000000000001322 26771796 PMC6486399

[20] Ozer A Ozcan S : Anesthetic neurotoxycity and neuroplasticity in pediatric patients. *Curr. Top. Anesthesiol.* 2017;1–10. 10.5772/65921

[21] Ellen McCann M Soriano SG : General anesthetics in pediatric anesthesia: influences on the developing brain. *Curr. Drug Targets.* 2012;13:944–951. 10.2174/138945012800675768 22512394 PMC4172352

[22] Beck K Arumuhum A Veronese M : N-methyl-D-aspartate receptor availability in first episode psychosis: a PET-MR brain imaging study. *Trans. Psychiatry.* 2021;11(425):1–8. 10.1038/s41398-021-01540-2 PMC8361127 34385418

[23] Kotani Y Shimazawa M Yoshimura S : The experimental and clinical pharmacology of propofol, an anesthetic agent with neuroprotective properties. *CNS Neurosci. Ther.* 2008;14:95–106. 10.1111/j.1527-3458.2008.00043.x 18482023 PMC6494023

[24] Yusriyani TA : Effect of intravenous propofol administration on hippocampal caspase 3 expression in balb/c mice with head injury. *J. Neuroanestesi. Indones.* 2013;2:81–88.

[25] Hutagalung TR : Effect of administration of turmeric extract (curcuma domestica val.) on the expression of mature brain derived neurotrophic factor (m-BDNF) in male rats Sprague-Dawley strain after traumatic brain injury. Tesis, Universitas Sumatera Utara. 2018.

[26] Zhou J Wang F Zhang J : The interplay of BDNF-TrkB with NMDA receptor in propofol induced cognition dysfunction. *BMC Anesthesiol.* 2018;18(35):1–8. 10.1186/s12871-018-0491-y 29621970 PMC5887174

[27] Lin PH Kuo LT Luh HT : The roles of neurotrophins in traumatic brain injury. *Life.* 2022;12:1–22. 10.3390/life12010026 PMC8780368 35054419

[28] Juananda D Cahyani D Sari R : Effects of chronic stress on the brain: biomolecular study of glucocorticoid hormones and post-regulation Brain-Derived Neurotrophic Factor (BDNF) in the Cerebellum. *FK UGM.* 2015;9:65–70. 10.26891/JIK.v9i2.2015.65-70

[29] Wu A Ying Z Pinilla FG : Dietary Omega-3 Fatty Acids Normalize BDNF Levels, Reduce Oxidative Damage and Counteract Learning Disability after Traumatic Brain Injury in Rats. *J. Neurotrauma.* 2004;21(10):1457–1467. 10.1089/neu.2004.21.1457 15672635

[30] Elfving B Plougmann PH Wegener G : Detection of brain-derived neurotrophic factor (BDNF) in rat blood and brain preparations using ELISA: Pitfalls and solutions. *J. Neurosci. Methods.* 2010;187(1):73–77. 10.1016/j.jneumeth.2009.12.017 20043947

[31] Hicks RR Li C Zhang L : Alterations in BDNF and trkB mRNA levels in the cerebral cortex following experimental brain trauma in rats. *J. Neurotrauma.* 2019;16:501–510. 10.1089/neu.1999.16.501 10391366

[32] Nazwar TA Balafif F Wardhana DW : Potential of Nigella Sativa on Anti or Pro-apoptotic Pathway After Brain Injury Via Brain-derived Neurotrophic factor on Rattus Norvegicus Wistar. *Teikyo Med. J.* 2023;46(01):7683–7696.

[33] Motaghinejad M Motevalian M Fatima S : Curcumin confers neuroprotection against alcohol-induced hippocampal neurodegeneration via CREB-BDNF pathway in rats. *Biomed. Pharmacother.* 2017;87:721–740. 10.1016/j.biopha.2016.12.020 28095363

[34] Wu A Ying ZHE Gomez-pinilla F : Dietary Omega-3 Fatty Acids Normalize BDNF Levels, Reduce Oxidative Damage, and Counteract Learning Disability after Traumatic Brain Injury in Rats. *J. Neurotrauma.* 2004;21:1457–1467. 10.1089/neu.2004.21.1457 15672635

[35] Oliveira CO Ikuta N Regner A : Outcome Biomarkers Following Severe Traumatic Brain Injury. *Rev. Bras. Ter. Intensiva.* 2008;20(4):411–421. 10.1590/S0103-507X2008000400015 25307248

[36] Jacquens A Needham EJ Zanier ER : Neuro-inflammation modulation and post-traumatic brain injury lesions: from bench to bed-side. *Int. J. Mol. Sci.* 2022;23(19):11193. 10.3390/ijms231911193 36232495 PMC9570205

[37] Hausburg MA Banton KL Roman PE : Effects of propofol on ischemia-reperfusion and traumatic brain injury. *J. Crit. Care.* 2020;56:281–287. 10.1016/j.jcrc.2019.12.021 32001426

[38] White TE Ford GD Surles-Zeigler MC : Gene expression patterns following unilateral traumatic brain injury reveals a local pro-inflammatory and remote anti-inflammatory response. *BMC Genomics.* 2013 Apr 25;14:282. 10.1186/1471-2164-14-282 23617241 PMC3669032

[39] Xi HJ Zhang TH Tao T : Propofol improved neurobehavioral outcome of cerebral ischemia-reperfusion rats by regulating Bcl-2 and Bax expression. *Brain Res.* 2011 Sep 2;1410:24–32. 10.1016/j.brainres.2011.06.060 21783180

[40] Shi SS Yang WZ Chen Y : Propofol reduces inflammatory reaction and ischemic brain damage in cerebral ischemia in rats. *Neurochem. Res.* 2014 May;39(5):793–799. 10.1007/s11064-014-1272-8 24610527

[41] Zhou R Yang Z Tang X : Propofol protects against focal cerebral ischemia via inhibition of microglia-mediated proinflammatory cytokines in a rat model of experimental stroke. *PLoS One.* 2013 Dec 9;8(12):e82729. 10.1371/journal.pone.0082729 24349350 PMC3857282

[42] Kulsum K : BDNF Dataset.[Dataset]. In F1000research. *Zenodo.* 2023. 10.5281/zenodo.10276418

[43] Kulsum K : ARRIVE checklist for research entitled: Effect of ethanol extract of nigella sativa L seeds and propofol on BDNF protein level as neuroplasticity and neuroprotection of traumatic brain injury in rats. In F1000Research. *Zenodo.* 2023. 10.5281/zenodo.10276505 PMC11729193 39810851

