# Supplementary material for: Effect of ethanol extract of nigella sativa L seeds and propofol on BDNF protein level as neuroplasticity and neuroprotection of traumatic brain injury in rats
Source: F1000Res. 2024 Sep 23;13:275. Originally published 2024 Apr 15. [Version 2] doi: 10.12688/f1000research.142054.2 (PMC11729193; doi:10.12688/f1000research.142054.2)
Supplement: Peer review history

# Reviewer report: Reviewer response for version 2

**DOI:** 10.5256/f1000research.171974.r354944

**Published:** 2025-01-13

**Authors:** Tommy Nazwar

**Affiliations:** 1 Universitas Brawijaya, Malang, East Java, Indonesia

This article presents an intriguing and relevant topic on the effects of *Nigella sativa* and propofol on BDNF protein levels as indicators of neuroplasticity and neuroprotection in a traumatic brain injury model using rats. The study holds significant potential to contribute to the development of new therapeutic strategies for patients with traumatic brain injury. However, several aspects of the methodology, data analysis, and interpretation of results require further clarification to enhance the validity and clinical relevance of the findings.

1. Why did the authors choose the "Feeney weight drop" method for modeling traumatic brain injury? Does this method most accurately represent the clinical conditions of traumatic brain injury in humans, particularly concerning the role of BDNF?
2. Were the doses of *Nigella sativa* (500 mg/kgBW) and propofol (10 mg/kgBW) selected based on previous studies demonstrating optimal neuroprotective effects, or were preliminary experiments conducted to determine these doses?
3. Are there plans or additional data to evaluate the long-term effects of *Nigella sativa* and propofol administration on neuroplasticity, including potential side effects or the animals' tolerance to these treatments?
4. In the context of clinical application, have the authors considered differences in the metabolism of *Nigella sativa* and propofol between rats and humans? How can the findings from this study be translated to inform therapeutic strategies for traumatic brain injury in human patients?
5. Has there been further investigation into the potential pharmacological interactions between *Nigella sativa* and propofol, particularly at the molecular level, such as modulation of the BDNF pathway or additive effects on neuroplasticity?
6. How do the authors correlate the observed increase in BDNF levels with histopathological findings in the brain? Have the presumed neuroprotective mechanisms been confirmed through measurable structural changes in the brain observed histologically?

I hope these suggestions help improve and refine this manuscript.

Is the work clearly and accurately presented and does it cite the current literature?

Yes

If applicable, is the statistical analysis and its interpretation appropriate?

Yes

Are all the source data underlying the results available to ensure full reproducibility?

Yes

Is the study design appropriate and is the work technically sound?

Yes

Are the conclusions drawn adequately supported by the results?

Partly

Are sufficient details of methods and analysis provided to allow replication by others?

Partly

Reviewer Expertise:

Neurotrauma, Neuroprotection, and Translational Neuroscience

I confirm that I have read this submission and believe that I have an appropriate level of expertise to confirm that it is of an acceptable scientific standard, however I have significant reservations, as outlined above.

# Reviewer report: Reviewer response for version 2

**DOI:** 10.5256/f1000research.171974.r326142

**Published:** 2024-09-27

**Authors:** Mohamad Sofyan Harahap

**Affiliations:** 1 Diponegoro University,, Semarang, Indonesia

The author has made revisions according to our directions and in general the article is suitable for indexing.

Is the work clearly and accurately presented and does it cite the current literature?

Yes

If applicable, is the statistical analysis and its interpretation appropriate?

Yes

Are all the source data underlying the results available to ensure full reproducibility?

Partly

Is the study design appropriate and is the work technically sound?

Yes

Are the conclusions drawn adequately supported by the results?

Partly

Are sufficient details of methods and analysis provided to allow replication by others?

Yes

Reviewer Expertise:

stress , inflammation and brain protection

I confirm that I have read this submission and believe that I have an appropriate level of expertise to confirm that it is of an acceptable scientific standard.

# Reviewer report: Reviewer response for version 1

**DOI:** 10.5256/f1000research.155549.r315110

**Published:** 2024-09-03

**Authors:** Mohamad Sofyan Harahap

**Affiliations:** 1 Diponegoro University,, Semarang, Indonesia

Overall, the author has a good idea and will enrich the view of brain protection. The author has tried to find a simple method with easily obtained materials, and made a comparison with Propofol which is considered by researchers as the gold standard, because Propofol is used clinically in the fields of anesthesia and intensive care.

 In developing new materials (Nigella Sativa extract), it is correct to use animal trials, with the rules that have been attempted properly, but the technique of administering Propofol to mice must be in accordance with the correct method so that it can be reworked by other researchers.

 Likewise, regarding the model for creating artificial head injuries, the method must be clear so that it can be replicated by other researchers.

 I think the two things above are my major corrections that need to be clarified by the author.

 My suggestion to help improve this research and hopefully increase the quality of the research results is the use of anatomical pathology examination of the brains of mice that have been euthanized, so that it can be assessed pathologically with the right method, the level of damage or protective effects of the two test materials, (Propofol and Nigella Sativa).

Is the work clearly and accurately presented and does it cite the current literature?

Yes

If applicable, is the statistical analysis and its interpretation appropriate?

Yes

Are all the source data underlying the results available to ensure full reproducibility?

Partly

Is the study design appropriate and is the work technically sound?

Yes

Are the conclusions drawn adequately supported by the results?

Partly

Are sufficient details of methods and analysis provided to allow replication by others?

Yes

Reviewer Expertise:

stress , inflammation and brain protection

I confirm that I have read this submission and believe that I have an appropriate level of expertise to confirm that it is of an acceptable scientific standard, however I have significant reservations, as outlined above.

# Response

**Published:** 2024-09-16

**Authors:** Syahrul Syahrul

**Affiliations:** Universitas Syiah Kuala, Indonesia

Dear: Prof. **Mohamad Sofyan Harahap**, Diponegoro University, Semarang, Indonesia

 Thank you very much for your generous comment. I was greatly helped by your suggestions, and I revised our article manuscript according to your suggestions.

**Reviewer comment: **The technique of administering Propofol to rats must be in accordance with the correct method so that it can be reworked by other researchers.

**Author response: **We agree with the suggestion, here we add information in the study design and subjects’ section, namely propofol is an anesthetic and intensive therapy drug, for the technique of administering propofol is by intravenous injection or continuous syringe pump. In this study, propofol was given to the tail of the rat because there are many blood vessels there so that it can quickly reach the systemic blood circulation, in addition, oxygen saturation monitoring was carried out through pulse oximetry because the side effect of propofol is falling asleep until apnoea so that oxygen saturation monitoring here is to prevent oxygen desaturase.

**Reviewer comment: **Likewise, regarding the model for making artificial head injuries, the method must be clear so that it can be replicated by other researchers.

**​​​​​​​Author response: **We agree with the suggestion. We will add information in the administration of TBI section, in this study, artificial head injuries were carried out using the Feeney weight drop mode. The stages of this method are sequential, first, the Rattus norvegicus head hair is shaved then given local anaesthesia to reduce pain in the animal model, then a 4 mm incision is made above the right frontoparietal bone area. Second, the centre of the scalp was then opened at a position of 1.5 mm from the posterior part of the bregma and 2.5 mm from the lateral midline. Third, Rattus norvegicus was dropped from a height of 1 meter with the head down, the part of the head that was exposed was the right frontoparietal bone.

**Reviewer comment: **To help improve this research and hopefully improve the quality of research results is to utilize pathological anatomical examination of the brains of euthanized rats, so that it can be assessed pathologically with the right method, the level of damage or protective effects of the two test materials, (Propofol and Nigella Sativa).

**Author response:** We strongly agree with your suggestion, we have done this from another side of this research, namely by examining the effects of Propofol and Nigella Sativa on the histopathological picture of post-traumatic brain injury as an indicator of neuroprotection.

 Best regards

 Prof.Syahrul

# Reviewer report: Reviewer response for version 1

**DOI:** 10.5256/f1000research.155549.r293532

**Published:** 2024-07-15

**Authors:** Li Liu

**Affiliations:** 1 Harbin Medical University, Harbin, Heilongjiang, China

The title of this article is "Effect of ethanol extract of *Nigella sativa* L seeds and promotion on BDNF protein level as neuroplasticity and neuroprotection of traumatic brain injury in rates", which is an interesting study. This article uses advanced statistical methods to discover the advantageous effects of ethanol extract from *Nigella sativa* L seeds in traumatic brain injury. However, there are several aspects that need to be addressed before indexing:

 1. This article only uses BDNF as an indicator for observation. Although the role of BDNF in neuroprotection is well-known, it has not been reflected in the neuroprotection and neuroplasticity aspects of this experiment.

 2. Propofol is a commonly used anesthetic in clinical anesthesia and intensive care units. In this study, it is unreasonable for clinical application that is continuous intravenous infusion of traumatic rats for 7 days.

 3. Why is there no statistical significance between the G1 group and the G2 group in Table 6? This is very confusing.

Is the work clearly and accurately presented and does it cite the current literature?

Yes

If applicable, is the statistical analysis and its interpretation appropriate?

Yes

Are all the source data underlying the results available to ensure full reproducibility?

Yes

Is the study design appropriate and is the work technically sound?

Yes

Are the conclusions drawn adequately supported by the results?

Partly

Are sufficient details of methods and analysis provided to allow replication by others?

Yes

Reviewer Expertise:

Perioperative brain protection

I confirm that I have read this submission and believe that I have an appropriate level of expertise to confirm that it is of an acceptable scientific standard, however I have significant reservations, as outlined above.

# Response

**Published:** 2024-08-10

**Authors:** Syahrul Syahrul

**Affiliations:** Universitas Syiah Kuala, Indonesia

Dear Prof. **Li Liu**, 

 Harbin Medical University, Harbin, Heilongjiang, China

 Thank you very much for your generous comment. I was greatly helped by your suggestions, and I revised our article manuscript according to your suggestions.

1. In the introductory section, we have provided information about the benefits of BDNF as an indicator of neuroprotection, as well as neuroplasticity, in which section it has been mentioned that BDNF is an important molecule in neuroplasticity. In our study, the effects of Nigella sativa and Propofol were tested using the ELISA method to determine the effects of neuroplasticity and neuroprotection by observing the increase in BDNF protein levels. In this study, neuroplasticity was reflected in the increase in BDNF protein levels, while neuroprotection was reflected in the average BDNF protein levels.
2. We agree with your view that propofol has been widely used in anaesthesia services and ICU management. We need to add information that: In this study, propofol was not given intravenously for 7 days but was given intravenously only once at the beginning of the study so as not to cause a prolonged hypoxic head effect.
3. Thank you for your correction. We agree to add information about the level of significance between groups G1 and G2 to the narrative at the end of the results section. In Table 6, it is listed, but not narrated. Here is the additional information: Group G1 differs on average above G2 by 49.10350 points but is not statistically significantly different between G1 and G2 (p = 0.122, p > 0.05).Thus sequentially the difference in average BDNF protein levels from the five groups is G5>G3>G4>G1>G2.

Best regards

 Syahrul
